# Cardiovascular Disease Risk Factors in Ghana during the Rural-to-Urban Transition: A Cross-Sectional Study

**DOI:** 10.1371/journal.pone.0162753

**Published:** 2016-10-12

**Authors:** Nuri Kodaman, Melinda C. Aldrich, Rafal Sobota, Folkert W. Asselbergs, Kwabena A. Poku, Nancy J. Brown, Jason H. Moore, Scott M. Williams

**Affiliations:** 1 Department of Genetics, Geisel School of Medicine, Dartmouth College, Hanover, New Hampshire, United States of America; 2 Vanderbilt Genetics Institute, Vanderbilt University Medical School, Nashville, Tennessee, United States of America; 3 Department of Thoracic Surgery and Division of Epidemiology, Vanderbilt University Medical School, Nashville, Tennessee, United States of America; 4 Department of Cardiology, Division Heart & Lungs, UMC Utrecht, Utrecht, the Netherlands; 5 Durrer Center for Cardiogenetic Research, ICIN-Netherlands Heart Institute, Utrecht, the Netherlands; 6 Institute of Cardiovascular Science, Faculty of Population Health Sciences, University College London, London, United Kingdom; 7 University of Ghana, Legon, Ghana; 8 Department of Medicine, Vanderbilt University Medical School, Nashville, Tennessee, United States of America; 9 Department of Biostatistics and Epidemiology, The Perelman School of Medicine, University of Pennsylvania, Philadelphia, Pennsylvania, United States of America; 10 Department of Epidemiology and Biostatistics, Case Western Reserve University, Cleveland, Ohio, United States of America; Loyola University Chicago, UNITED STATES

## Abstract

Populations in sub-Saharan Africa are shifting from rural to increasingly urban. Although the burden of cardiovascular disease is expected to increase with this changing landscape, few large studies have assessed a wide range of risk factors in urban and rural populations, particularly in West Africa. We conducted a cross-sectional, population-based survey of 3317 participants from Ghana (≥18 years old), of whom 2265 (57% female) were from a mid-sized city (Sunyani, population ~250,000) and 1052 (55% female) were from surrounding villages (populations <5000). We measured canonical cardiovascular disease risk factors (BMI, blood pressure, fasting glucose, lipids) and fibrinolytic markers (PAI-1 and t-PA), and assessed how their distributions and related clinical outcomes (including obesity, hypertension and diabetes) varied with urban residence and sex. Urban residence was strongly associated with obesity (OR: 7.8, 95% CI: 5.3–11.3), diabetes (OR 3.6, 95% CI: 2.3–5.7), and hypertension (OR 3.2, 95% CI: 2.6–4.0). Among the quantitative measures, most affected were total cholesterol (+0.81 standard deviations, 95% CI 0.73–0.88), LDL cholesterol (+0.89, 95% CI: 0.79–0.99), and t-PA (+0.56, 95% CI: 0.48–0.63). Triglycerides and HDL cholesterol profiles were similarly poor in both urban and rural environments, but significantly worse among rural participants after BMI-adjustment. For most of the risk factors, the strength of the association with urban residence did not vary with sex. Obesity was a major exception, with urban women at particularly high risk (26% age-standardized prevalence) compared to urban men (7%). Overall, urban residents had substantially worse cardiovascular risk profiles, with some risk factors at levels typically seen in the developed world.

## Introduction

Urban populations in the developing world are growing rapidly and at an accelerating rate[1]. Rural-to-urban transitions are often associated with marked changes in behavior and lifestyle, such as diminished physical activity, sedentary employment, poorer dietary habits, and increased psychosocial stress[2]. In part because of these emerging risk factors, over 80% of the global burden of cardiovascular disease (CVD) has now shifted to low- and middle-income countries[3]. While proper screening and preventive strategies have reduced CVD in high-income countries, individuals at risk in the developing world are much less likely to be identified and treated, because of poor infrastructure, inadequate resources, and a lack of awareness regarding CVD and its symptoms in general[4].

The fastest rate of urbanization worldwide is occurring in sub-Saharan Africa, driven by high fertility rates and rapid industrialization[1]. The transition from pre-industrial to industrialized economies has initiated an epidemiological transition from illnesses related to malnutrition, childbirth, and infection, towards chronic, non-communicable diseases, such as CVD[5]. However, the epidemiological transition in sub-Saharan Africa is still in its early stages. As a consequence, diseases such as HIV and malaria continue to strain limited resources and dominate the public consciousness, while CVD and its often-subclinical symptoms are overlooked[6]. Thus, populations are becoming older and more vulnerable to CVD at a time when surveillance capacities remain poor and skilled health workers scarce[6, 7].

Our knowledge of CVD epidemiology in sub-Saharan Africa is incomplete[8]. Early surveys (pre-1990) revealed that risk factors such as hypertension and diabetes were rare, fueling the hypothesis that CVD is not of substantial public health interest[9–12]. More recently, this view has begun to change[7, 13, 14]. Nonetheless, variation in study designs and the diversity of the populations being studied have generated an often-confusing picture[13, 15, 16]. While some reports suggest that the proportion of disease burden attributed to CVD in sub-Saharan Africa may still be relatively low (primarily on account of persistent infectious disease-related mortality), the average age of death from CVD is the youngest in the world[17]. Thus, all the makings of a CVD epidemic are in place, as both life expectancy and urban populations increase.

Much of our understanding of CVD risk is based on studies of European populations, despite the fact that both the prevalence of risk factors and their relation to CVD endpoints differ among ethnic groups[18, 19]. Existing risk assessment algorithms, such as the Framingham score, may consequently be prone to error when applied globally. Moreover, while such algorithms are typically calculated separately for males and females[20], the effect of sex on CVD incidence and risk profile can also vary with culture and ethnicity[21]. Indeed, sex-specific effects appear to be more pronounced in the developing world, perhaps owing to differences in cultural practices and social behavior[22–24].

Given these heterogeneities of CVD risk profiles by sex, environment, and population, a multifactorial approach to CVD assessment and intervention is essential. Here, we describe how major CVD risk factors, including dyslipidemia, hypertension, obesity, and diabetes, are distributed among urban and rural Ghanaian men and women from a single ethnic group. In addition to the conventional CVD risk factors, we also assess plasma levels of two fibrinolytically active enzymes that may provide deeper insight into CVD risk and pathophysiology [25], plasminogen activator inhibitor-1 (PAI-1) and tissue plasminogen activator (t-PA). PAI-1 impedes the removal of thrombi from the vascular system by binding to and neutralizing t-PA’s thrombolytic properties, such that high circulating PAI-1 increases the risk of thromboembolic events [26, 27], while also playing a role in atherosclerosis[28]. Our overriding goal is to evaluate the prevalence of CVD risk factors in the region and to understand some of the conditions that may give rise to them, establishing a baseline for future comparisons and setting guidelines for appropriate recommendations.

## Materials and Methods

### Study Population

Participants were recruited from Sunyani, the capital of the Brong Ahafo region of Ghana, population 250,000 as of the 2012 census, and from 31 surrounding rural villages of fewer than 5000 people within a 10 km radius of the city. Urban recruitment for the study began in 2002 and ended in 2007[29]. Rural participants were all recruited in 2008, from randomly selected households and household members. Participants had all results explained to them in small groups or one-on-one and were given a free clinical consultation if measurements were well above normal. Few potential participants refused to engage. Urban participants learned about the study at public venues, including local churches and markets. Exclusion criteria included acute illness, age <18 years, or first or second degree relation to someone already enrolled in the study. Questionnaires were filled out by study personnel who sat with the participants until the forms were completed (**S1 File**). Information obtained included medical history, current medications, and demographic and socio-economic data, such as education level and smoking status. All participants provided informed consent in writing. Institutional review boards at Vanderbilt University, Dartmouth College, and Regional Hospital, Sunyani approved all protocols.

### Anthropometric measurements and biochemical analyses

Standing height and weight were measured to calculate body mass index (BMI). Blood pressures were measured using an Omron HEM-705c instrument (Omron Healthcare Corp., Bannockburn, Illinois, USA). Two measurements were taken from the left arm of participants after they were seated in a quiet location for 10 minutes. The mean of the two measurements for both systolic blood pressure (SBP) and diastolic blood pressure (DBP) were used in statistical analyses. In the case when the two measurements differed by more than 10 mmHG, a third measurement was taken and the outlier removed. In addition, all measurements were taken between the hours of 8 AM and 10 AM by only 3 people during the entire study.

Blood was drawn between 8:00 and 10:00 AM, after ≥8 hour fast, and used to assess fasting glucose, fasting lipids, and t-PA/PAI-1 levels. Glucose was measured with a SureStep monitor by LifeScan (Milpitas, California, USA), using blood drops from the blood-draw needles. Total cholesterol (TC), triglycerides (TG) and high-density lipoprotein cholesterol (HDL) levels were measured in plasma using an Elan ATAC 8000 Random Access Chemistry System (Elan Diagnostics, Smithfield, Rhode Island, USA); low-density lipoprotein cholesterol (LDL) levels were calculated using the Friedewald equation. Glucose and lipids were measured at the hospital where recruitment was performed. The missing HDL-C and LDL-C data for 1098 participants (588 urban, 510 rural) resulted from a supply failure for HDL cholesterol assay during the collection process. Because the supply shortages occurred sporadically and randomly, there is unlikely to be any systematic difference between participants with and without complete data. PAI-1 and t-PA were measured at Vanderbilt University in the same laboratory after shipment in liquid nitrogen dry-shippers, using enzyme-linked immunoassay (Biopool AB, Umea).

### Categorical outcomes

Hypertension was defined as: SBP ≥140 mm Hg, DBP ≥90 mm Hg, or current use of antihypertensive medication prescribed by a physician[30, 31]. Diabetes was defined as a fasting glucose level ≥126 mg/dL or current use of an antidiabetic medication prescribed by a physician.[32] Impaired fasting glucose (IFG) represents an intermediate state of abnormal glucose regulation, associated with abnormal glucose tolerance, and often termed “pre-diabetes.” The American Diabetes Association (ADA) now defines IFG as fasting glucose ≥100 mg/dL, having lowered the threshold from ≥110 mg/dL in 2003[33], whereas the World Health Organization (WHO) continues to recommend the 110 mg/dL cut point, citing a lack of evidence that lowering it offers any benefit with respect to reducing adverse outcomes[34]. All analyses below were performed using both thresholds, and are referred to accordingly.

Total cholesterol (TC), low-density lipoprotein cholesterol (LDL), and triglycerides (TG) were considered high if they were ≥200 mg/dL, ≥130 mg/dL, ≥110 mg/dL, respectively; while high-density lipoprotein cholesterol (HDL) was considered low ≤40 mg/dL[35–38]. Generally, a higher threshold (50 mg/dL) is used to determine low HDL in women than in men (40 mg/dL)[39]. However, a primary focus of this study was to understand how risk factor outcomes varied with sex, and in this regard, using different definitions for men and women would have made relevant comparisons asymmetric, unnecessarily complicating conclusions. The lower (more conservative) threshold was therefore used for both sexes. However, the age-standardized prevalence of low HDL in urban and rural women was also estimated using the higher HDL threshold.

Obesity was defined as BMI ≥30 kg/m^2^, while BMI ≥25 kg/m^2^ was deemed overweight (and therefore includes obesity)[40]. All participants who smoked in the last 30 days qualified as current smokers. Years of education were dichotomized into two variables, one reflecting whether a participant had any formal schooling and the other education beyond Junior Secondary School (JSS). Ghanaian students typically attend JSS until age 15 in preparation for the “Basic Education Certificate Examination”[41, 42].

### Statistical Methods

Crude means and standard deviations or, where appropriate, medians and interquartile ranges were calculated for all continuous variables after participants were stratified by sex and urban/rural environment into groups: urban males (UM), urban females (UF), rural males (RM), and rural females (RF). Fasting glucose, TG, t-PA, PAI-1, and the TC-to-HDL ratio were log transformed to obtain normal or near-normal distributions. All mean comparisons were performed after age-adjustment. Mean comparisons between sexes stratified by residence (UM vs. UF, RM vs. RF) and between urban and rural residents stratified by sex (UM vs. RM, UF vs. RF) were performed using *t*-tests that allowed for unequal variances. Urban-rural differences in sex-adjusted means and male-female differences in residence-adjusted means were standardized (using pooled standard deviations of residuals) to estimate the “effect sizes” of urban environment and sex, respectively, on CVD risk factors [43]. These analyses were also performed after adjustment for BMI. Our use of the term “effect size” does not imply any conclusions about actual cause or direction of effect, but rather should be taken as shorthand for the difference in an outcome that is associated with a specified factor, in standardized units. Effect sizes associated with “education beyond JSS” on CVD risk factors was also assessed, using age- and sex-adjusted residuals; this analysis was not performed on rural residents as too few had such schooling to be included. The “rules of thumb” for interpreting standard mean differences are as follows: 0.2 standard deviation = small effect; 0.5 = moderate effect; and 0.8 = large effect[43]. To estimate the odds ratios of clinical outcomes with respect to environment or sex, similar analyses using logistic regression models were used after controlling for sex and environment, respectively. Prevalence estimates of clinical outcomes were standardized according to the WHO 2000–2025 standard population, using recommended age bins that pertained to our data (18–24, 25–34, 35–44, 45–54, ≥55 years-old)[44, 45]. Mean values of all categorical and continuous variables were also calculated separately for these age groups. Statistical analyses were performed using STATA (version 12) and JMP (version 11).

## Results

Of the 3317 individuals that met eligibility criteria, 2265 (68%) were urban dwellers (57% female) and 1293 rural (55% female). Ages ranged from 18–99 and were similarly distributed among urban males (UM), urban females (UF), rural males (RM), and rural females (RM) (p = 0.23, Kruskal-Wallis test), with medians of 42.5, 43.5, 42, and 42, respectively (**Table 1**). Smoking was extremely rare among UF (0%), RF (2%) and UM (3%). The 16% of RM who qualified as smokers generally did not smoke cigarettes, but rather their own leaves, presumably tobacco (**Table A in S2 File**). Almost all UM (96%) and a similarly large proportion of UF (88%) reported some formal education (**Table A in S2 File**). Although this was true for only 64% of RM and 44% of RF, the difference was strongly related to age cohort (**Figure A in S2 File**). For education beyond JSS, the contrast between urban and rural was even greater, with 48% and 30% of UM and UF meeting the criterion, but only 5% of RM and 2% of RF (**Table A** and **Figure A in** **S2 Fi**le).

**Table 1:** Physiologic and metabolic variables in the Ghanaian cohort.

	Females			Males			Urban	Rural
	Urban	Rural	p-value	Urban	Rural	p-value	p-value by sex	p-value by sex
N	1293	583		972	469			
**Age (years)**	42.1 (11.3)	43.9 (15.9)	0.005	42.9 (12.6)	44.9 (17.2)	0.005	0.113	0.333
**BMI (kg/m**^**2**^**)**	26.9 (5.6)	22.9 (3.9)	<0.001	24.0 (3.9)	21.5 (2.7)	<0.001	<0.001	<0.001
**SBP (mm Hg)**	125.1 (18.3)	123.8 (20.2)	0.002	130.2 (18.9)	127.3 (16.9)	0.002	<0.001	<0.001
**DBP (mm Hg)**	77.7 (10.7)	73.7 (11.6)	<0.001	78.0 (12.4)	73.5 (10.8)	<0.001	0.694	0.623
**TC (mg/dL)**	181.8 (42.1)	152.3 (36.9)	<0.001	170.6 (42.5)	142.2 (36.4)	<0.001	<0.001	<0.001
**LDL-C (mg/dL)**	113.9 (37.6)^1^	88.6 (32.3)^2^	<0.001	106.3 (34.1)^3^	76.4 (27.3)^4^	<0.001	<0.001	<0.001
**HDL-C (mg/dL)**	49.2 (14.6)^1^	46.5 (15.9)^2^	0.002	43.5 (13.3)^3^	44.5 (14.7)^4^	0.002	<0.001	0.212
**TC/HDL-C**	3.8 (1.6)^1^	3.3 (1.8)^2^	<0.001	3.9 (1.7)^3^	3.2 (1.5)^4^	<0.001	<0.001	0.097
**TG (mg/dL)**	77 (47)	82 (52)	0.103	83 (57)	82.5 (53)	0.103	<0.001	0.084
**Glucose (mg/dL)**	93 (15)	94 (14)	0.371	91 (15)	90 (14)	0.371	<0.001	<0.001
**t-PA (ng/mL)**	6.4 (4.6)	4.3 (3.4)	<0.001	6.7 (5.3)	5.6 (4.3)	<0.001	0.004	<0.001
**PAI-1 (ng/mL)**	3.9 (6.3)	2.9 (4.4)	<0.001	3.7 (6.3)	3.5 (4.8)	<0.001	0.282	0.253

^1^n = 955

^2^n = 317

^3^n = 722

^4^n = 225

Data shown as: crude mean (standard deviation), except for TC/HDL-C, TG, glucose, t-PA, and PAI-1, shown as: median (interquartile range); BMI: body mass index; SBP: systolic blood pressure; DBP: diastolic blood pressure; TC: total cholesterol; LDL-C: low density lipoprotein cholesterol; HDL-C: high density lipoprotein cholesterol; TG: triglycerides; Glucose: fasting plasma glucose; t-PA—tissue plasminogen activator; PAI-1: plasminogen activator inhibitor

p-value: *t*-test (allowing for unequal variances) was performed on age-adjusted residuals to evaluate significance of difference between means; TC/HDL, TG, glucose, t-PA, and PAI-1 were first log-transformed.

### Blood Pressure and Hypertension

**Fig 1:**
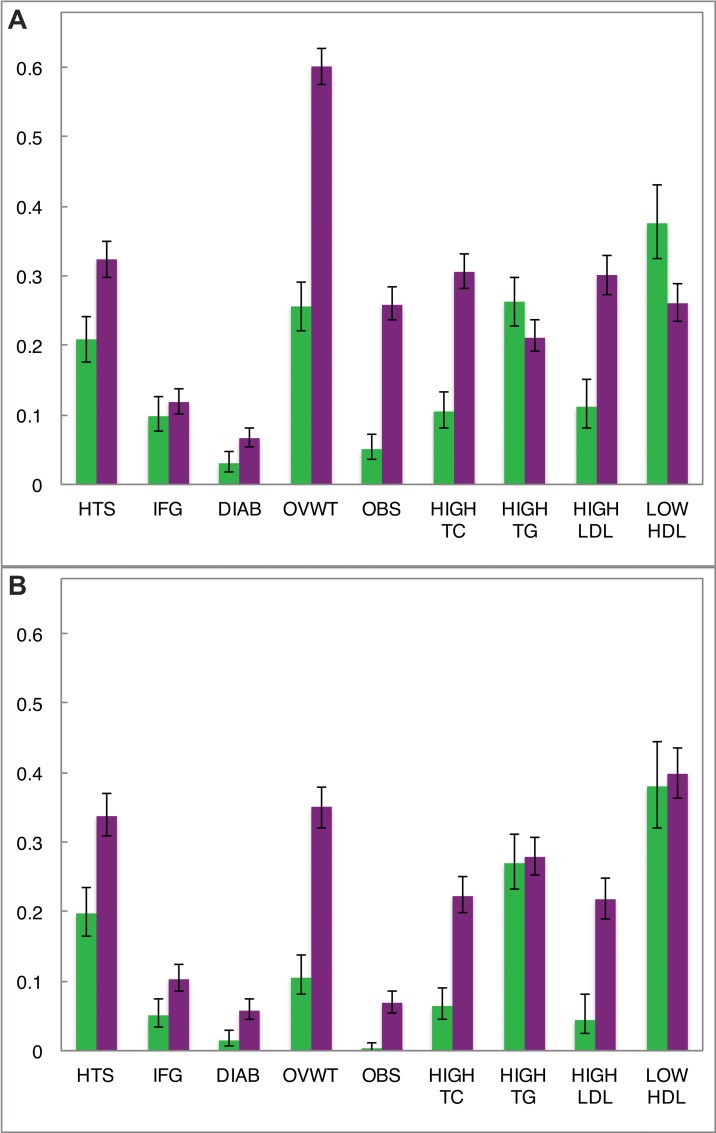
Age-standardized prevalence of dichotomous clinical outcomes by sex and urban/rural environment in Brong Ahafo, Ghana. (A) Urban (purple) and rural (green) females; (B) urban (purple) and rural (green) males. Error bars denote 95% confidence intervals of estimates. HTS: hypertension (SBP ≥140 or DBP ≥90); IFG: impaired fasting glucose (using the WHO cut-point of 110 mg/dL); DIAB: diabetes (glucose ≥126 mg/dL); OVWT: overweight (BMI ≥25); OBS: obesity (BMI ≥30); HIGH TC: hypercholesterolemia (cholesterol ≥200 mg/dL); HIGH TG: elevated triglycerides (≥ 110 mg/dL); HIGH LDL: elevated low-density lipoprotein cholesterol (≥130 mg/dL); LOW HDL: low high-density lipoprotein cholesterol (≤40 mg/dL). For UF, RF, UM, and RM, N = 1293, 583, 972, and 469 (except for HIGH LDL and LOW HDL: N = 955, 317, 722, 225), respectively. All data age-standardized to the WHO 2000–2025 standard population.

**Fig 2:**
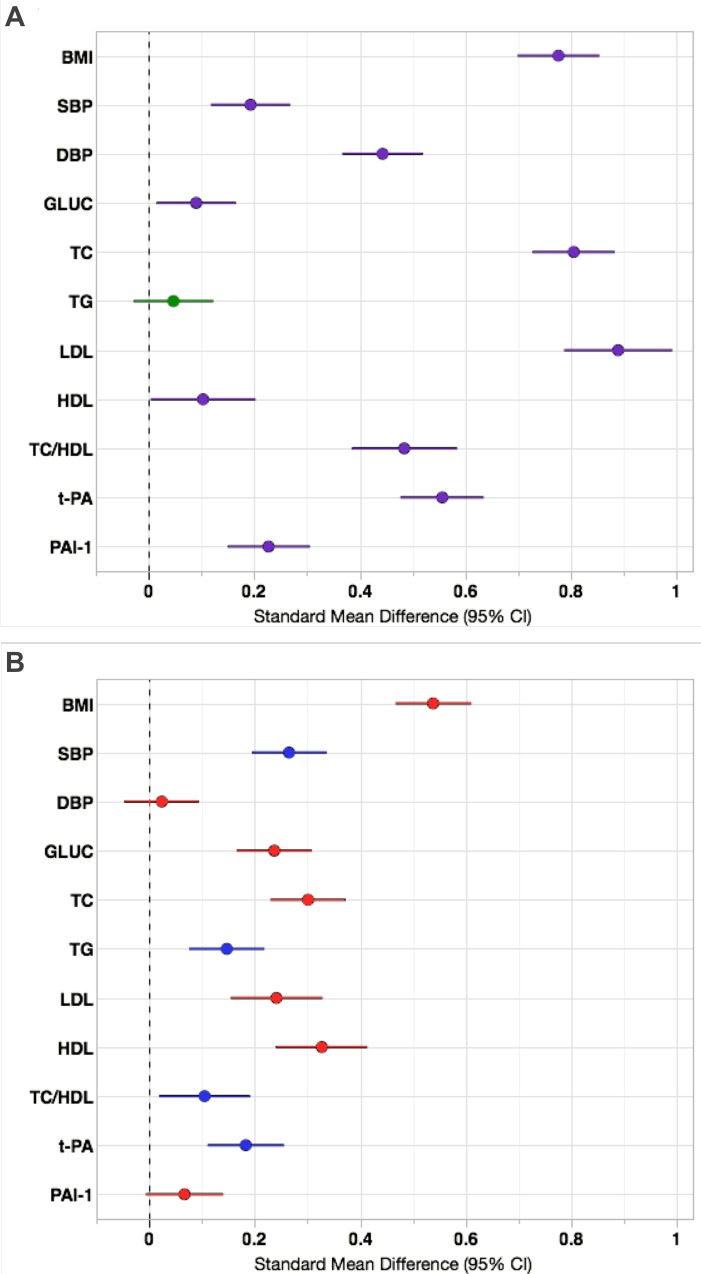
Effect sizes of urban/rural environment and sex on continuous cardiovascular risk factors in Brong Ahafo, Ghana. (A) Absolute differences between urban and rural standardized means (with 95% confidence intervals); purple lines indicate that the urban group has a higher mean and green lines indicate that the rural group has a higher mean. Data were adjusted for age and sex. (B) Absolute differences between male and female standardized means (with 95% confidence intervals); red lines indicate that the females have a higher mean and blue lines indicate that males have a higher mean. Data were adjusted for age and urban/rural residence. Abbreviations as described in methods.

**Fig 3:**
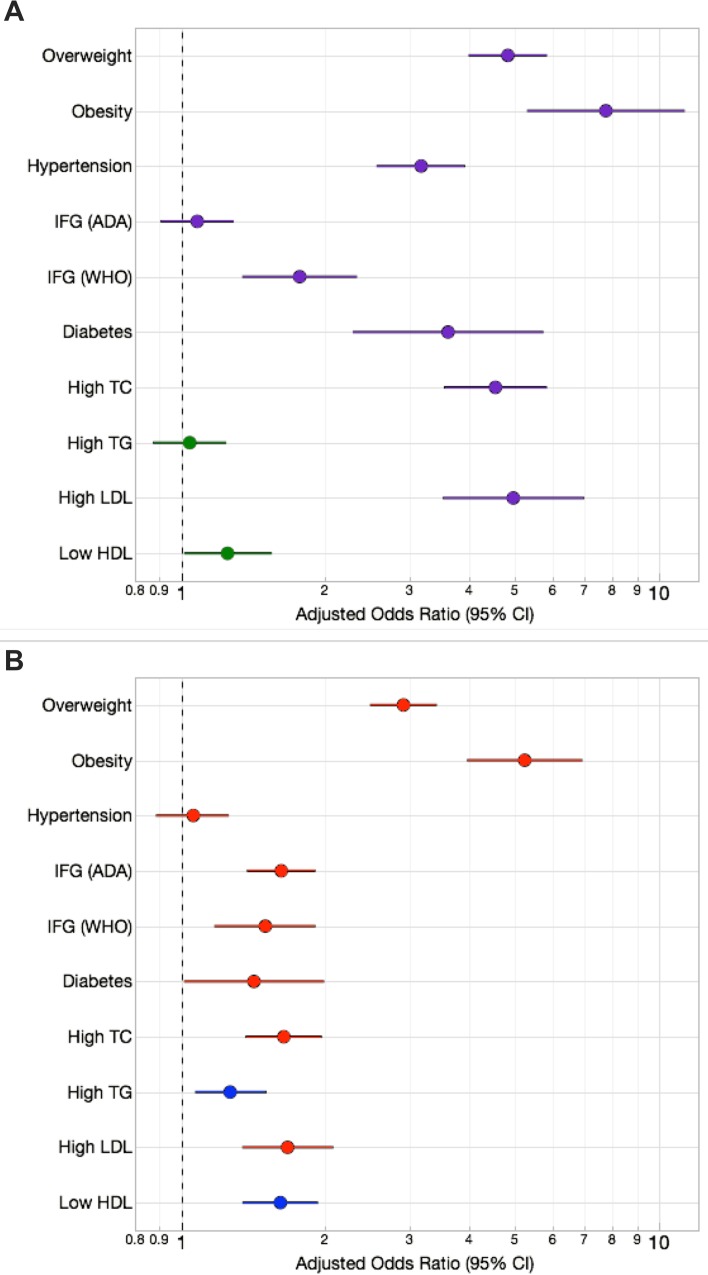
Effect sizes of urban/rural environment and sex on dichotomous cardiovascular risk factors in Brong Ahafo, Ghana. (A) The increased odds of each outcome (with 95% confidence intervals) are depicted for the group with the higher odds; purple for higher urban odds and green for higher rural odds. Data were adjusted for age and sex. (B) The increased odds of each outcome (with 95% confidence intervals) are depicted for the group with the higher odds (female: red; male: blue). Data were adjusted for age and environment. Abbreviations as described in methods.

**Fig 4:**
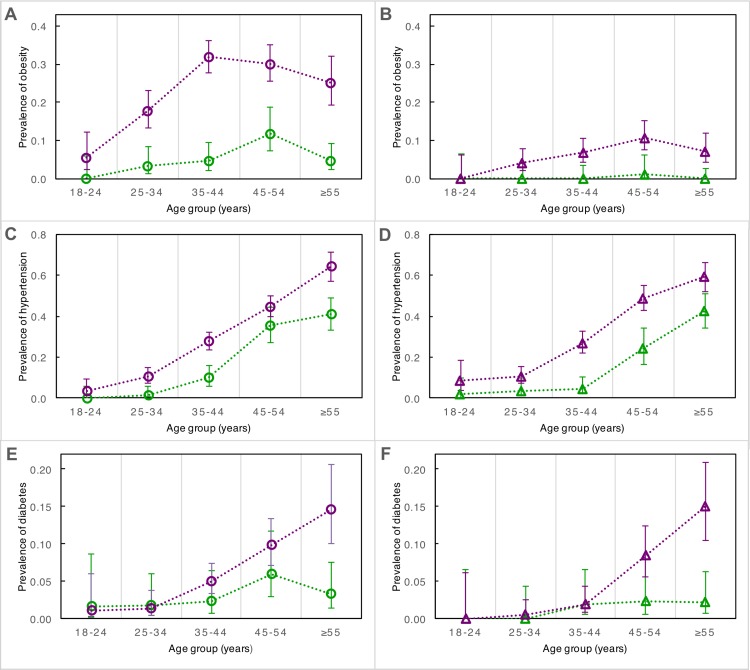
Prevalence by age group of obesity, hypertension, and diabetes in urban and rural men and women from Brong Ahafo, Ghana. In the left panels (A), (C), and (E), prevalence by age group is depicted for urban females (purple circles) and rural females (green circles). In the right panels (B), (D), and (F), prevalence by age group is depicted for urban males (purple triangles) and rural males (green triangles). Error bars denote 95% confidence intervals of estimates.

In within-sex analyses (UM vs. RM, UF vs. RF), SBP, DBP, and the prevalence of hypertension were significantly greater in the urban cohort (**Table 1** and **Fig 1**; **Table A in S2 File**). In comparisons between sexes (UM vs. UF, RM vs. RF), only SBP differed significantly, and was higher in men (p<0.001) (**Table 1**; **Table A in S2 File**). Male sex and urban environment had “small” effect sizes on SBP, while urban environment had a “moderate” effect size on DBP and hypertension (see Methods for criteria; **Figs 2** and **3**). The marked increase in blood pressure and hypertension that corresponded with age began about a decade earlier in the urban cohort than the rural (**Fig 4**; **Figure B in S2 File**).

### BMI, Overweight and Obesity

Mean BMI, obesity, and the prevalence of overweight status (“overweight”) were significantly greater among urban residents (stratified by sex) and among women (stratified by residence) (**Table 1** and **Fig 1; Table A in S2 File**). The effect size of urban residence on sex-adjusted BMI was large, while that of female sex was moderate (**Fig 2**). This pattern was exaggerated at the right tail of the BMI distribution, with the odds of being overweight (BMI ≥25) or obese (BMI ≥30) 4.8 and 7.6 times greater, respectively, among urban residents (**Fig 3A**). Females had 2.9 times greater odds of being overweight and 5.2 times greater odds of being obese than males (**Fig 3B**). In a logistic regression model of obesity as a function of age, sex and residence, adding a sex-by-residence interaction term improved the model significantly (p<0.0001), reflecting the much higher prevalence of obesity in urban women. By age 45, rates of overweight and obesity among urban women were 70% and 35%, respectively (**Fig 4**; **Figure C in S2 File**).

### Fasting Glucose, Impaired Fasting Glucose, and Diabetes

Age-standardized prevalence of diabetes was significantly greater among urban participants of both sexes (6.6% for UF, 5.7% for UM) (**Fig 1**; **Table A in S2 File**). Differences between sexes stratified by residence were not significant (**Fig 1**; **Table A in S2 File**). Differences in fasting glucose and IFG prevalence (using the ADA’s 100 mg/dL cut-point), on the other hand, were consistently significant only between sexes (**Table 1** and **Fig 1**; **Table A in S2 File**). The effect size of sex on fasting glucose was small, while that of urban residence was even smaller (by roughly half) (**Fig 2**). However, urban residents had 3.6 times greater odds of diabetes (**Fig 3**). Urban residence was only a risk factor for IFG, or “pre-diabetes,” when the WHO glucose threshold was used. With the ADA threshold urban and rural residents had roughly the same odds of IFG. Mean fasting glucose was positively associated with age in all groups, whereas a sharp increase in diabetes was evident only among UF (≥35 years old) and UM (≥45 years old) (**Fig 4**; **Figure D in S2 File**).

### Lipid traits and Dyslipidemias

**Fig 5:**
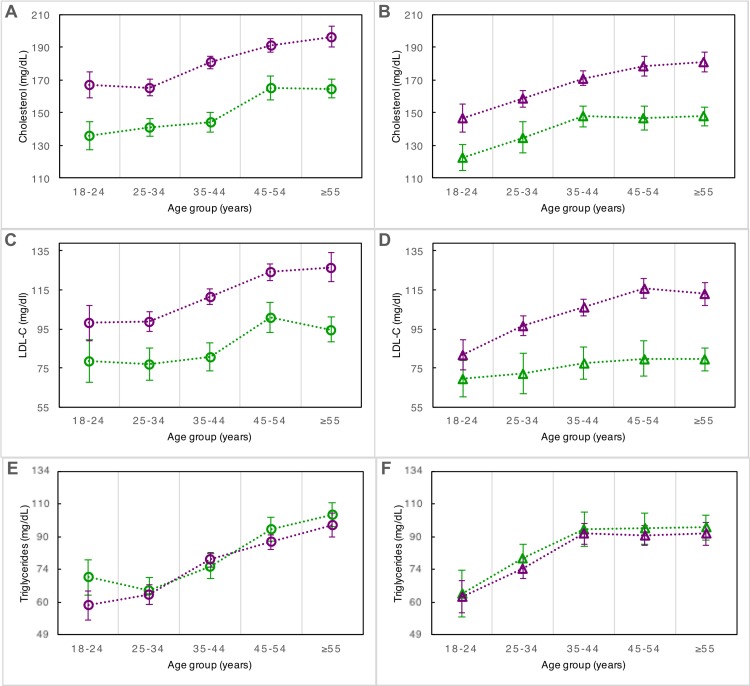
Mean lipid levels by age group in urban and rural men and women from Brong Ahafo, Ghana. In the left panels (A), (C), and (E), mean estimates by age group are depicted for urban females (purple circles) and rural females (green circles). In the right panels (B), (D), and (F), mean estimates by age group are depicted for urban males (purple triangles) and rural males (green triangles). Error bars denote 95% confidence intervals. Note: in (E) and (F), vertical axis is logarithmic.

TC and LDL were significantly higher in urban males and females than in their rural counterparts, as was TC/HDL (**Table 1**). Adjusted for sex, the standardized effect size of urban environment on TC and LDL was almost one standard deviation, and on TC/HDL, approximately one-half standard deviation. However, urban residence was not significantly associated with either TG or HDL (**Table 1, Fig 2A**). The effect size on TC and LDL was robust to adjustment for BMI, whereas TG and HDL profiles became significantly worse in rural residents after such adjustment (**Figure E in S2 File**). Female sex had a small deleterious effect size on TC and LDL, and a small beneficial one on HDL (**Fig 2B**). Results were broadly similar for analyses on dyslipidemias as dichotomous traits (**Figs 1** and **3**). Regarding the diagnosis of low HDL, when the 40 mg/dL threshold was used, the age-standardized prevalence was 26% and 40% in urban men and women, respectively, and 38% in both rural men and women (**Table A in S2 File**). When a higher threshold of 50 mg/dL was used to diagnose low HDL in urban and rural women, the age-standardized rates of prevalence increased to 58% (95% CI: 55–62%) and 61% (95% CI: 56%-66%), respectively. TC, LDL, and TG increased similarly with age regardless of environment (**Fig 5**), while HDL was the only trait in this study that did not associate with age (**Figure F in S2 File**). Among urban residents, education beyond JSS was highly associated with hypercholesterolemia (p<0.0001), but only weakly associated with overweight and hypertension (p = 0.01 and 0.05, respectively), and not significantly associated with increased obesity or diabetes.

### PAI-1 and t-PA

All four pairwise comparisons of mean t-PA (UM vs. RM, UF vs. RF, UM vs. UF, and RM vs. RF) were significant, while only one of four tests yielded significant results for PAI-1 (UF vs. RF) (**Table 1**). The effect sizes of sex and urban residence on t-PA were small and moderate, respectively. Sex did not associate with PAI-1, while urban residence had a small effect size (**Fig 2**). The pattern of higher t-PA with increasing age was similar for all groups, whereas PAI-1 was more variable (**Figure G in S2 File**).

## Discussion

Economic development in sub-Saharan Africa has fostered an epidemiological transition, marked by an increase in the burden of chronic diseases, including cardiovascular disease. In Ghana, where more than half of the population now live in urban areas, recent reports indicate a rise in the prevalence of conditions such as hypertension, diabetes, and obesity[13, 15, 46, 47]. Here we have surveyed a number of different cardiovascular risk factors to present a relatively broad view of cardiovascular disease risk in the region, both as it currently stands and as we may expect it to increase with continued urbanization.

Once considered virtually absent in the sub-Saharan African region, hypertension has quickly emerged as a major epidemic[48–50]. In our study population, the age-standardized prevalence among urban residents (33%) was in the upper range of estimates previously reported for West African cities, including Accra, the capital of Ghana (30%)[13, 46, 51]. Also in keeping with previous reports, hypertension prevalence did not differ by sex[13]. Urban residents were at significantly greater risk than rural residents, likely because their mean DBP was ~0.5 standard deviation greater. In contrast, the difference in SBP was minor. Although few large studies have assessed hypertension prevalence in rural West African populations, our estimates of 20% prevalence for rural men and 21% for rural women were similar to results from cross-sectional studies of similar rural populations[52–55]. The few instances where our estimates differ from those of prior studies may be explained by demographic differences between studies[56]. Taken together with previous studies[50, 57–60], our data therefore indicate that hypertension should no longer be considered rare in rural West Africa, where the absence of infrastructure makes timely detection unlikely.[61] Untreated, it is the primary cause of hemorrhagic stroke, the leading cause of CVD-related death in people of African descent.[17]

Awareness of diabetes throughout sub-Saharan Africa is also low, and undiagnosed cases common, such that affected individuals are at higher risk for complications than in the developed world[62]. In contrast to earlier studies in Ghana, which estimated the prevalence of diabetes at 0.2–0.4% [11, 12], our estimates for the urban Ghanaians were comparable to estimates for adults in the developed world[63]. Interestingly, although urban residents were significantly more likely to have diabetes than rural residents, they did not have higher fasting glucose levels. In fact, median fasting glucose levels were highest among rural females. This held even for study participants over the age of 55, when urban-rural diabetes prevalence diverged considerably. Thus, our results indicate that using only continuous or only dichotomous measurements may paint an incomplete picture of potentially meaningful differences between groups. The greater urban risk of diabetes was at least partly driven by the greater variance in fasting glucose among urban participants, though the possibility of joint effects with other correlated risk factors must also be considered.

The changes in lifestyle that accompany urbanization are unlikely to be uniform across sexes. Moreover, even when identical, exposures may affect men and women differently[64–68]. Thus, epidemiologic differences between sexes can be expected to reflect not only underlying pathophysiological and sociocultural factors, but also their interactions. Our results for BMI support this, as the combined effects of urban residence and female sex were greater than additive. The effect modifier is likely sociocultural[24, 69]. The 60% prevalence of overweight or obesity among urban women was comparable to the 64.9% prevalence reported by the Women’s Health Study of Accra. Prevalence of overweight and (particularly) obesity were extremely low among rural men and women, indicating that these conditions are driven almost entirely by factors related to urban residence. Therefore, the fact that obesity is increasing faster in Ghana than in any other West African nation can probably be attributed to the rapid rate of urbanization there relative to other nations in the region[70, 71]. Insofar as excessive adiposity and its associated comorbidities may impose costs on quality of life and health-care systems [72], this situation warrants strategies for prevention.

Few studies have assessed lipid traits in West Africa, and to our knowledge, no large study (N>1000) has done so in Ghana[73–75]. The relatively small number of African studies that have measured lipid traits have also reported generally low-risk profiles, creating the impression that dyslipidemia is not a health issue[76]. For example, a 2011 survey of serum total cholesterol in 199 countries and territories found sub-Saharan Africa to have the lowest mean level among all world regions (158 mg/dL)[75]. However, currently only about one-third of sub-Saharan Africans are living in urban areas. Because that number is rapidly increasing, with an inflection point projected for 2035[77], understanding the effects of urban residence on lipid profiles may be more important than estimating their present levels.

We found that urban residence had a stronger effect on age- and sex-adjusted TC and LDL than on any other risk factor. Remarkably, the urban/rural difference in TC appears to be driven almost entirely by differences in LDL, as there were no urban/rural differences in HDL or TG. The association of urban environment on TC and LDL was also robust to BMI adjustment, indicating that only some of the observed differences can be attributed to the BMI disparity. It is possible that, in addition to increasing adiposity, reduced physical activity also influences lipid traits directly [78]. Other factors related to urban lifestyle may also play a role, such as the composition of the diet. Interestingly, among urban residents, education beyond JSS was highly associated with hypercholesterolemia, but not significantly associated with increased obesity or diabetes.

Although the TC and LDL levels of rural participants were low, TG was unexpectedly high, and in fact, higher than that of urban participants. HDL profiles were also poor among rural participants, with levels ≤40 mg/dl in nearly 40% of men and women. In fact, when adjusted for BMI, the rural TG and HDL profiles were both significantly worse than the urban, indicating factors unique to the rural environment. Because HDL is a definitional component of TC, the low HDL levels of rural participants may be expected. Low HDL may be a risk factor regardless of TC[79, 80], but a recent study has called this into question[81]. However, the conjunction of low HDL with high TG, as reported here, may be particularly dangerous [82]. Although we have seen no explicit references to this trend, we note that other studies have also reported poor TG and HDL profiles in rural regions of other low- and middle-income countries, including India,[83] Nigeria,[84] Peru,[85] Mexico,[38] and Guatemala.[86] This phenomenon deserves further study.

The screening of risk factors for subclinical cardiovascular disease can help identify individuals at high risk of myocardial infarction and stroke.[25] We assessed two such non-traditional risk factors, PAI-1 and t-PA. Although t-PA and PAI-1 have opposite physiological roles, plasma t-PA is also positively correlated with CVD risk, because assays that measure t-PA typically detect it bound to PAI-1[87]. In our study, t-PA was more sensitive to both urban residence and sex than PAI-1. This may be of interest, as some prior results have indicated that t-PA is a better predictor of CVD risk than PAI-1[87]. However, the results for PAI-1 and t-PA were, to a large extent, directionally consistent, allowing us to conclude that urbanization is likely increasing cardiovascular risk because of pro-thrombotic and pro-inflammatory risk factors in addition to the conventional risk factors described above.

Although rural men had the healthiest cardiovascular profiles in this study, their PAI-1 levels were comparable to those of the urban participants. This was unexpected, because there was virtually no obesity among the rural men. In addition to being released by adipocytes, PAI-1 expression is also directly influenced by TG levels[88], which were high among rural men. However, there was no significant difference in TG levels between rural men and women (and rural women had the lowest PAI-1 levels among all groups). Thus, the reasons for high PAI-1 among rural men are not clear, although they appear to be sex-specific.

The limitations of our study pertain primarily to its cross-sectional design, which reduces our ability to elucidate causal relationships. Some of the prevalence differences we observed with age, for example, may have been driven by a birth cohort effect. Repeated measurements or secular trend data would be required to test this. Some selection bias may also have been introduced into our study, owing to our recruitment strategy in the city. However, because we sampled from a small city, the differences associated with urban residence presented here may actually be conservative. Furthermore, by sampling from rural villages of fewer than 5000 people where subsistence farming is still the main occupation, we have limited the potentially confounding factors introduced by technological advances into semi-rural settings. It is important to add that in our study we were not able to assess a variety of environmental factors that have previously been shown to associate with CVD, including physical activity, dietary differences, and psychological stress. Nonetheless, our study represents one of the largest comprehensive studies of its type in West Africa for a large array of CVD risk factors. Finally, our conclusions implicitly assumed that the risk factors we measured affect disease risk in African populations as they do in European populations (in whom most studies have been conducted).

Our results quantify the dramatic differences in CVD risk profiles associated with urban residence in Ghana. Urbanization appears to be the dominant factor in producing less favorable profiles related to blood pressure, BMI, fasting glucose, lipids, and PAI-1/t-PA. However, there were important exceptions, such as TG and HDL. While prospective studies in multiple venues will be required to clarify and build upon the results presented here, our findings indicate that continuing urbanization in the region is likely to impose a major public health burden in the years ahead.

## Supporting Information

S1 File The questionnaire used in this study to obtain information about medical history, smoking status, and demographic/socio-economic data. (PDF)

S2 File Collection of supporting tables and figures. (DOCX)
