# Supplementary material for: Cardiovascular Disease Risk Factors in Ghana during the Rural-to-Urban Transition: A Cross-Sectional Study
Source: PLoS One. 2016 Oct 12;11(10):e0162753. doi: 10.1371/journal.pone.0162753 (PMC5061429; doi:10.1371/journal.pone.0162753)
Supplement: S1 File — (PDF) [file pone.0162753.s001.pdf]

HEALTH INFORMATION
MEHARRY MEDICAL COLLEGE/UNIVERSITY OF GHANA

Date of Exam: __ __ / __ __ / 2 0 0 5
Month Day Year

Family ID: __ __ __ __ __ __ __

Identification Number: G H A T R __ __ __ __

Individual's Last Name: ____________

Individual's First Name: ____________

A1. ***ADDRESS***

City: ____________ **State:** __ __ **Zip Code:** __ __ __ __ __

Country (If not USA): G H A N A

Telephone Number (Including Area Code):

Home: ____________ **Work:** ____________

A2. ***SEX*** ☐ **Male** ☐ **Female**

A3. ***RACE*** ☐ **Black (of African origin)** ☐ **White (of European orgin)** ☐ **Other**

☐ **Tribe** **Please specify:** ____________

A4. ***DATE OF BIRTH*** **(MM/DD/YYYY):** __ __ / __ __ / __ __ __ __ **A5.** ***AGE*:** __ __

A6. ***In what country were you born?*** ____________

A7a. Mother's Last Name: ____________ **Mother's First Name:** ____________

A7b. What is your mother's tribe? ____________

A7c. Is she still alive? ☐ **Yes** ☐ **No** ☐ **Unknown**

A8a. Father's Last Name: ____________ **Father's First Name:** ____________

A8b. What is your father's tribe? ____________

A8c. Is he still alive? ☐ **Yes** ☐ **No** ☐ **Unknown**

A9. ***MARITAL STATUS:***

☐ **Married** ☐ **Living as Married** ☐ **Widowed** ☐ **Divorced** ☐ **Separated** ☐ **Never Married**

HEALTH INFORMATION (cont'd)

Family ID: ☐☐☐☐☐☐☐ **Identification Number:** G H A T R ☐☐☐☐

NUMBER OF CHILDREN

A10. Total: ☐☐ **A11. First Marriage:** ☐☐ **A14. Fourth Marriage:** ☐☐

A12. Second Marriage: ☐☐ **A15. Non-Marriage:** ☐☐

A13. Third Marriage: ☐☐

A16a. Do you have a TWIN sister or brother? ☐ Yes ☐ No

A16b. Is he/she participating in this study? ☐ Yes ☐ No ☐ Not Applicable

A17a. How many brothers do you have? ☐☐

(Please check "Half" for half brothers.)

	Last Names	First Names	Half	Age
1.			☐	
	How many years did you live with him? ☐☐ years	Is he still alive? Yes ☐ No ☐ Unk ☐		
2.			☐	
	How many years did you live with him? ☐☐ years	Is he still alive? Yes ☐ No ☐ Unk ☐		
3.			☐	
	How many years did you live with him? ☐☐ years	Is he still alive? Yes ☐ No ☐ Unk ☐		
4.			☐	
	How many years did you live with him? ☐☐ years	Is he still alive? Yes ☐ No ☐ Unk ☐		
5.			☐	
	How many years did you live with him? ☐☐ years	Is he still alive? Yes ☐ No ☐ Unk ☐		
6.			☐	
	How many years did you live with him? ☐☐ years	Is he still alive? Yes ☐ No ☐ Unk ☐		
7.			☐	
	How many years did you live with him? ☐☐ years	Is he still alive? Yes ☐ No ☐ Unk ☐		
8.			☐	
	How many years did you live with him? ☐☐ years	Is he still alive? Yes ☐ No ☐ Unk ☐		

Family ID: | | | | | | | |

Identification Number: G H A T R | | | | |

A17b. How many sisters do you have? | | |

(Please check "Half" for half sisters.)

	Last Names	First Names	Half	Age
1.			☐	
	How many years did you live with her? ____ years	Is she still alive? Yes ☐ No ☐ Unk ☐		
2.			☐	
	How many years did you live with her? ____ years	Is she still alive? Yes ☐ No ☐ Unk ☐		
3.			☐	
	How many years did you live with her? ____ years	Is she still alive? Yes ☐ No ☐ Unk ☐		
4.			☐	
	How many years did you live with her? ____ years	Is she still alive? Yes ☐ No ☐ Unk ☐		
5.			☐	
	How many years did you live with her? ____ years	Is she still alive? Yes ☐ No ☐ Unk ☐		
6.			☐	
	How many years did you live with her? ____ years	Is she still alive? Yes ☐ No ☐ Unk ☐		
7.			☐	
	How many years did you live with her? ____ years	Is she still alive? Yes ☐ No ☐ Unk ☐		
8.			☐	
	How many years did you live with her? ____ years	Is she still alive? Yes ☐ No ☐ Unk ☐		

A18. Education (***Highest Level***)

	# of Years
No School (enter "0")	
Primary	
Junior Secondary School	
Senior Secondary School	
Polytechnical School	
University/College	
Post Graduate	

A19. Occupation

- ☐ Farmer
- ☐ Trader
- ☐ Student
- ☐ Laborer (Unskilled)
- ☐ Craftsman (Skilled Labor) Specify:___________________________
- ☐ Office Worker
- ☐ Professional Specify:_________________________________
- ☐ Unemployed
- ☐ Other Specify:______________________________________

8540

Family ID: | | | | | | | |

Identification Number: G H A T R | | | | |

GENERAL HEALTH HISTORY

Has a doctor ever told you that you have had:

				AGE
B1. Heart attack (taken to hospital)	☐ Yes	☐ No	☐ Not Sure	
B2. Heart pain (angina, medication prescribed)	☐ Yes	☐ No	☐ Not Sure	
B3. Heart bypass surgery	☐ Yes	☐ No	☐ Not Sure	
B4. Stroke	☐ Yes	☐ No	☐ Not Sure	
B5. High blood pressure (medication prescribed)	☐ Yes	☐ No	☐ Not Sure	
B6. High blood cholesterol	☐ Yes	☐ No	☐ Not Sure	
B7. Diabetes	☐ Yes	☐ No	☐ Not Sure	
B8. If YES to B7, was insulin prescribed?	☐ Yes	☐ No	☐ Not Sure	
B9. Cancer (not skin cancer)	☐ Yes	☐ No	☐ Not Sure	

What kind? ____________

Because of high blood pressure have you ever taken medication:

B10. a) Prescription 1: ☐ Yes ☐ No

If **YES**, age when told: ____ Name of medication: ____________

Are you currently taking this medication? ☐ Yes ☐ No

If yes/no, how long ago did you start/stop taking it? ____ year(s) ____ month(s) ____ day(s)

B10. b) Prescription 2: ☐ Yes ☐ No

If **YES**, age when told: ____ Name of medication: ____________

Are you currently taking this medication? ☐ Yes ☐ No

If yes/no, how long ago did you start/stop taking it? ____ year(s) ____ month(s) ____ day(s)

B11. Are you currently taking any medication other than those above? ☐ Yes ☐ No

Name(s) of Current Medication:

1. ____________ For how long? ____ year(s) ____ month(s) ____ day(s)

2. ____________ For how long? ____ year(s) ____ month(s) ____ day(s)

3. ____________ For how long? ____ year(s) ____ month(s) ____ day(s)

4. ____________ For how long? ____ year(s) ____ month(s) ____ day(s)

8540

HEALTH INFORMATION (cont'd)

Family ID: ☐☐☐☐☐☐☐ **Identification Number:** G H A T R ☐☐☐☐

MEDICAL EXAM

Height: ☐☐☐ CM. **Weight:** ☐☐☐ . ☐☐ KGS.

Blood Pressure **1st Systolic (SBP):** ☐☐☐ (required) **1st Diastolic (DBP):** ☐☐☐ (required) **1st Pulse:** ☐☐☐ (required)

2nd Systolic (SBP): ☐☐☐ (optional) **2nd Systolic (DBP):** ☐☐☐ (optional) **2nd Pulse:** ☐☐☐ (optional)

TOBACCO USAGE

Smoker/Tobacco User: ☐ Yes ☐ No

Type of Tobacco:

☐ A)Cigarettes--------------------------------- Quantity (Cigarettes/Day): ☐☐☐☐ /Day

☐ B)Pipe-- Quantity (Pipes/Day): ☐☐☐☐ /Day

☐ C)Cigars--------------------------------------- Quantity (Number/Day): ☐☐☐☐ /Day

☐ D) Other types of tobacco ----- Type: ☐☐☐☐☐ Amount: ☐☐☐☐ /Day

SAMPLE INFORMATION

DNA: ☐ Yes ☐ No **Date Isolated:** ☐☐ / ☐☐ / ☐☐☐☐
Month Day Year

Storage Location: ☐☐☐☐☐☐☐☐☐☐☐☐☐☐☐☐☐☐☐☐

Plasma: ☐ Yes ☐ No **Date Isolated:** ☐☐ / ☐☐ / ☐☐☐☐
Month Day Year

Storage Location: ☐☐☐☐☐☐☐☐☐☐☐☐☐☐☐☐☐☐☐☐

METABOLIC PARAMETERS

Total Cholesterol ☐☐☐ . ☐ **mg/dl** **Triglycerides** ☐☐☐ . ☐ **mg/dl**

LDL ☐☐☐ . ☐ **mg/dl** **Fasting Glucose** ☐☐☐ . ☐ **mg/dl**

HDL ☐☐☐ . ☐ **mg/dl**

Notes:

8540
