# Supplementary material for: Cardiovascular Disease Risk Factors in Ghana during the Rural-to-Urban Transition: A Cross-Sectional Study
Source: PLoS One. 2016 Oct 12;11(10):e0162753. doi: 10.1371/journal.pone.0162753 (PMC5061429; doi:10.1371/journal.pone.0162753)
Supplement: S2 File — (DOCX) [file pone.0162753.s002.docx]

**S2 File. Supporting Table and Figures**

**
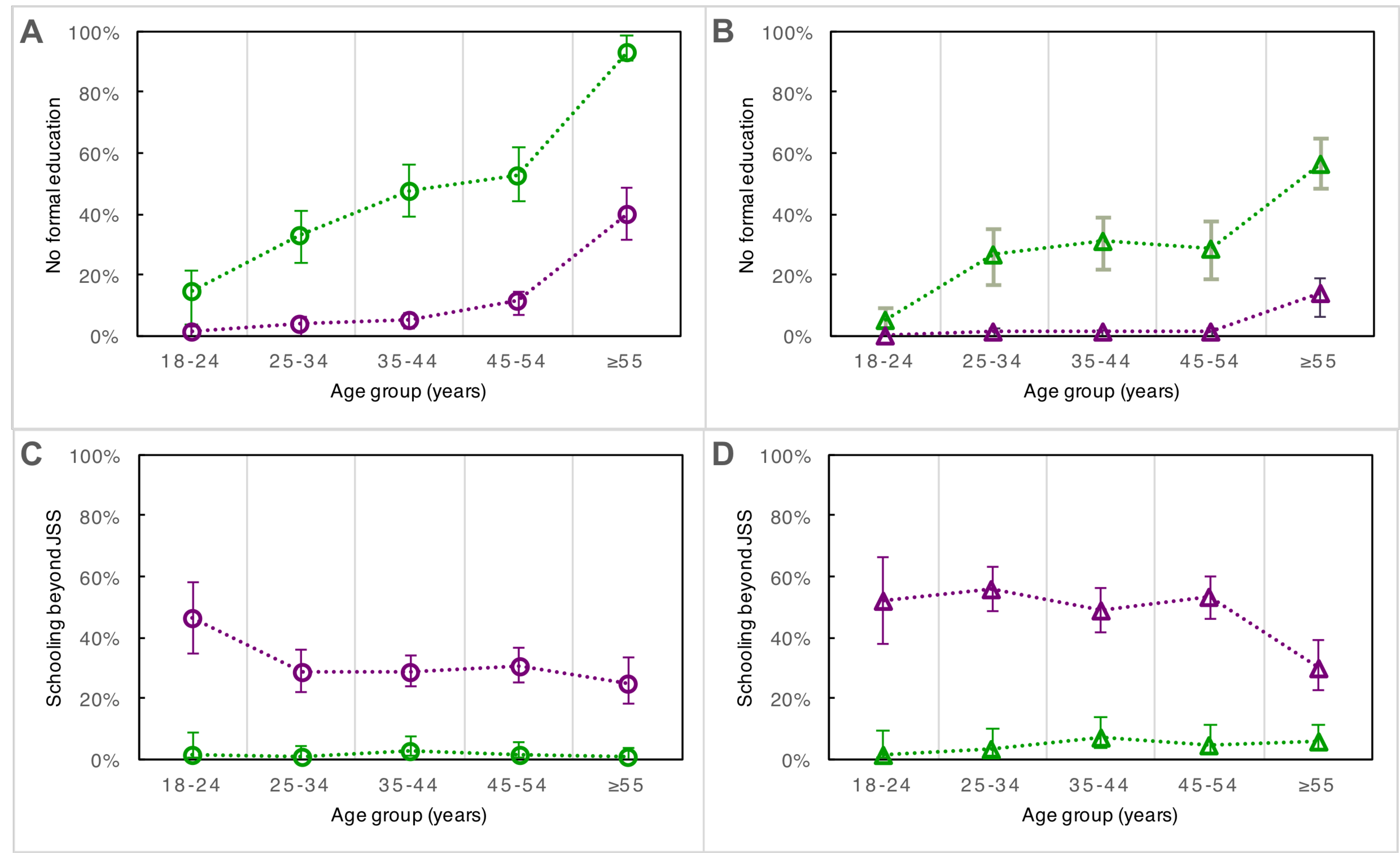
**

**Figure A.** **Education by age group among urban and rural men and women in Brong Ahafo, Ghana**. Left panels (A) and (C): estimates by age group are for urban females (purple circles) and rural females (green circles). Right panels (B) and (D): estimates by age group are for urban males (purple triangles) and rural males (green triangles). Error bars denote 95% confidence intervals. JSS = Junior Secondary School (usually attended through age 15).

**
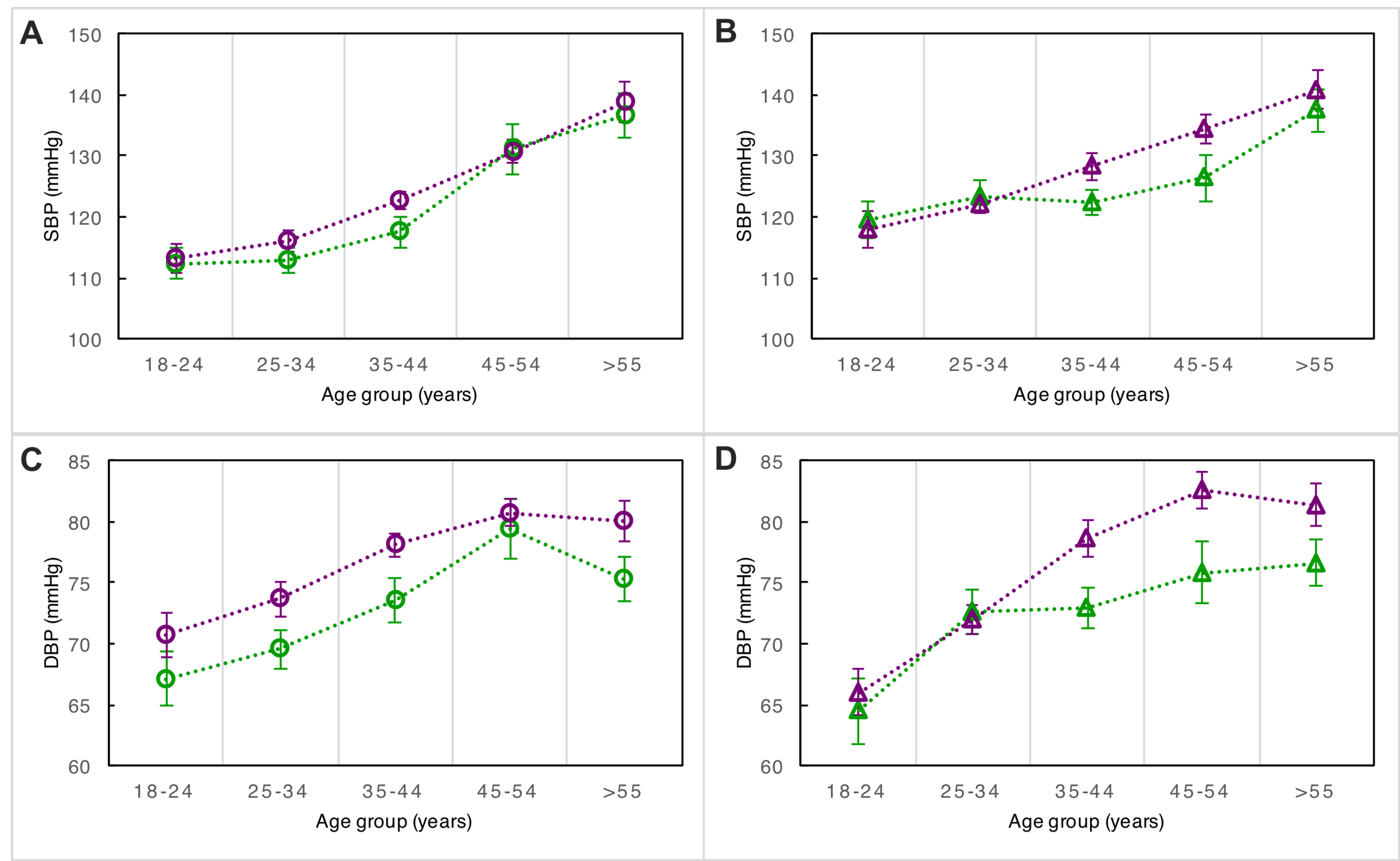
**

**Figure B.** **Mean systolic and diastolic blood pressure by age group in urban and rural men and women in Brong Ahafo, Ghana**. Left panels (A) and (C): mean estimates by age group for urban females (purple circles) and rural females (green circles). Right panels (B) and (D): mean estimates by age group for urban males (purple triangles) and rural males (green triangles). Error bars denote 95% confidence intervals.

**
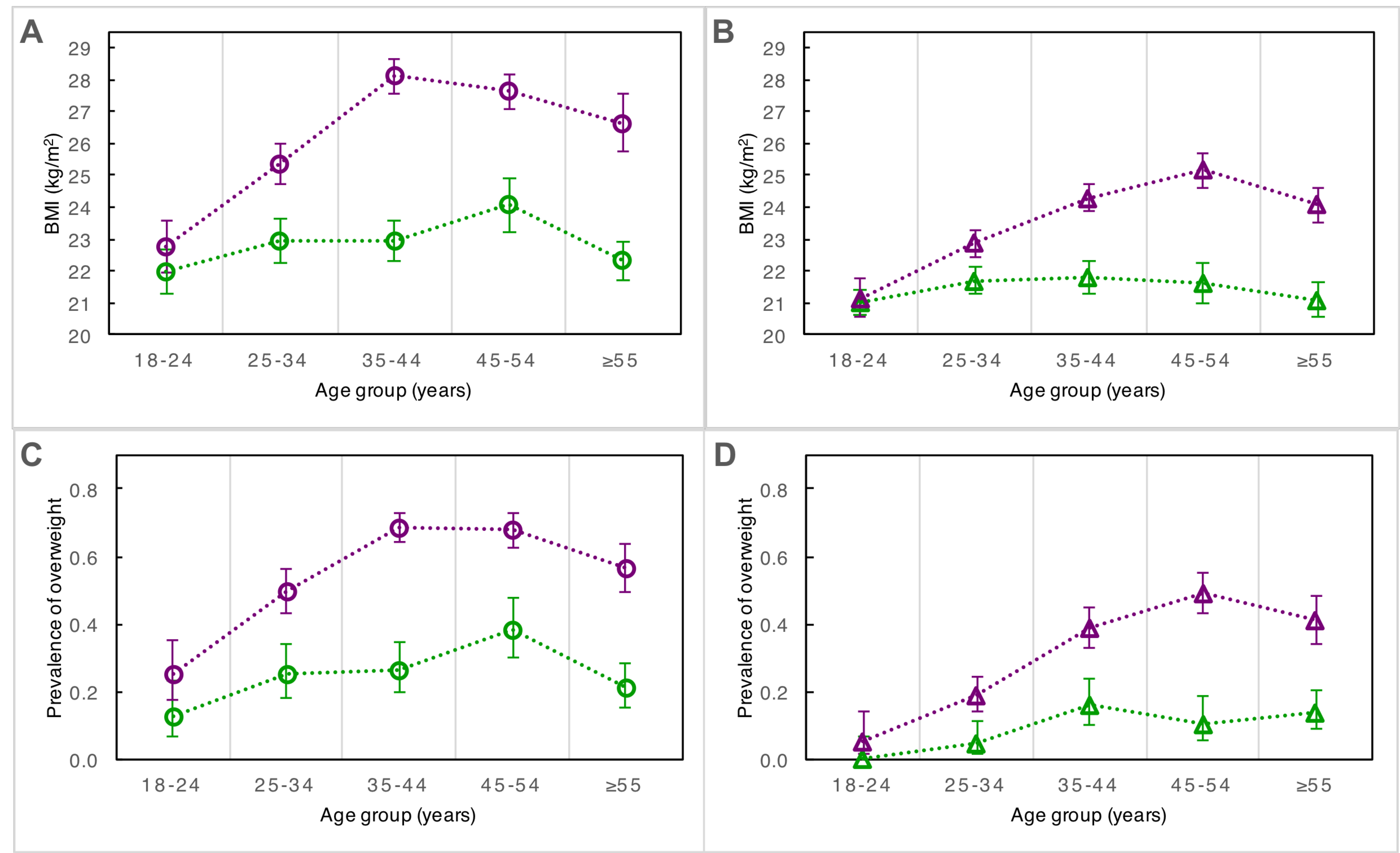
**

**Figure C.** **Mean BMI and overweight prevalence by age group in urban and rural men and women in Brong Ahafo, Ghana**. Left panels (A) and (C): estimates by age group for urban females (purple circles) and rural females (green circles). In the right panels (B) and (D), estimates by age group are depicted for urban males (purple triangles) and rural males (green triangles). Error bars denote 95% confidence intervals. Overweight is defined as BMI ≥ 25 kg/m^2^.

**
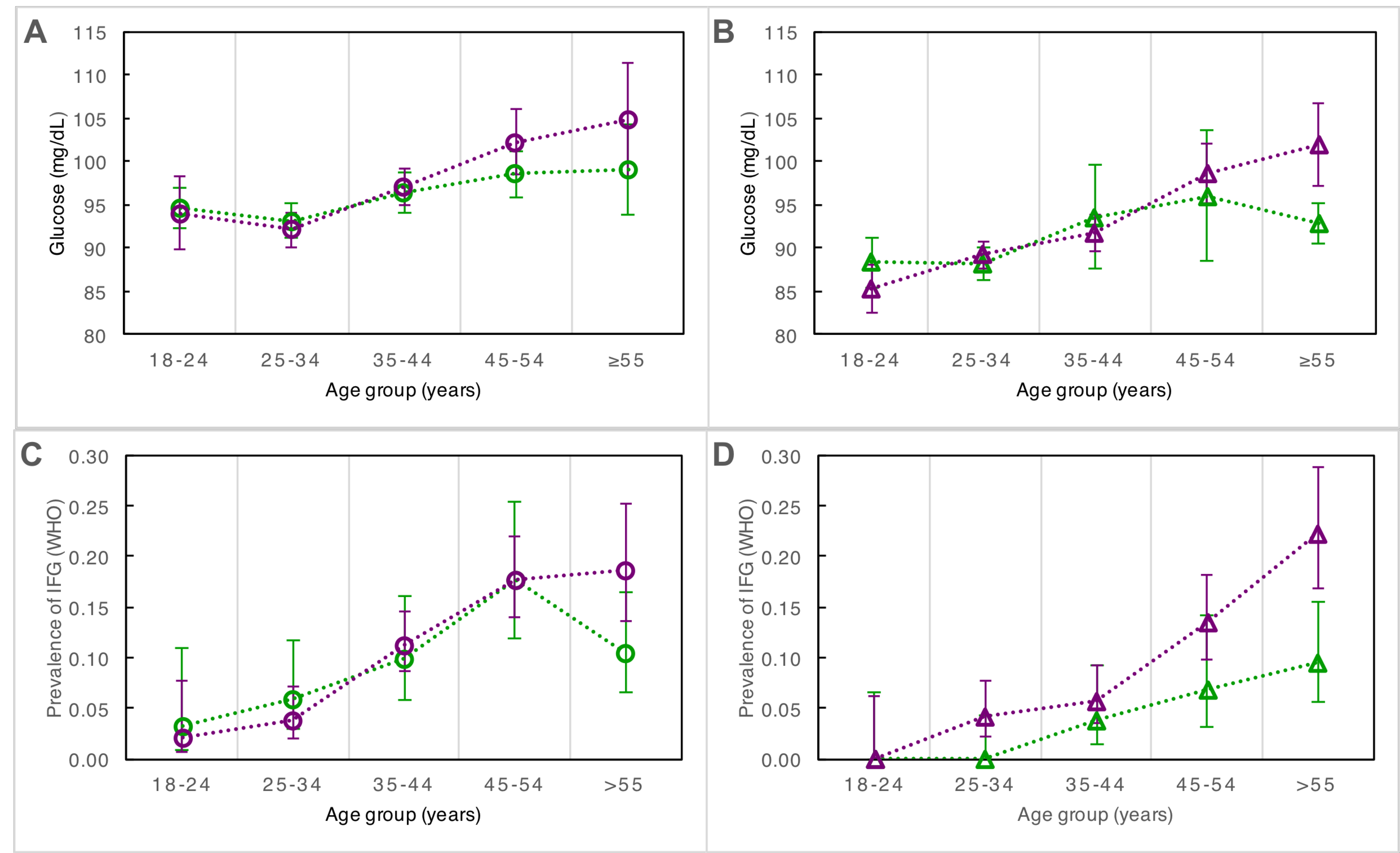
**

**Figure D.** **Mean fasting glucose and prevalence of impaired fasting glucose by age group in urban and rural men and women in Brong Ahafo, Ghana**. Left panels (A) and (C): urban females (purple circles) and rural females (green circles). Right panels (B) and (D): urban males (purple triangles) and rural males (green triangles). Error bars denote 95% confidence intervals. Impaired fasting glucose is defined according to the WHO cut-point (glucose ≥ 110 mg/dL).

**
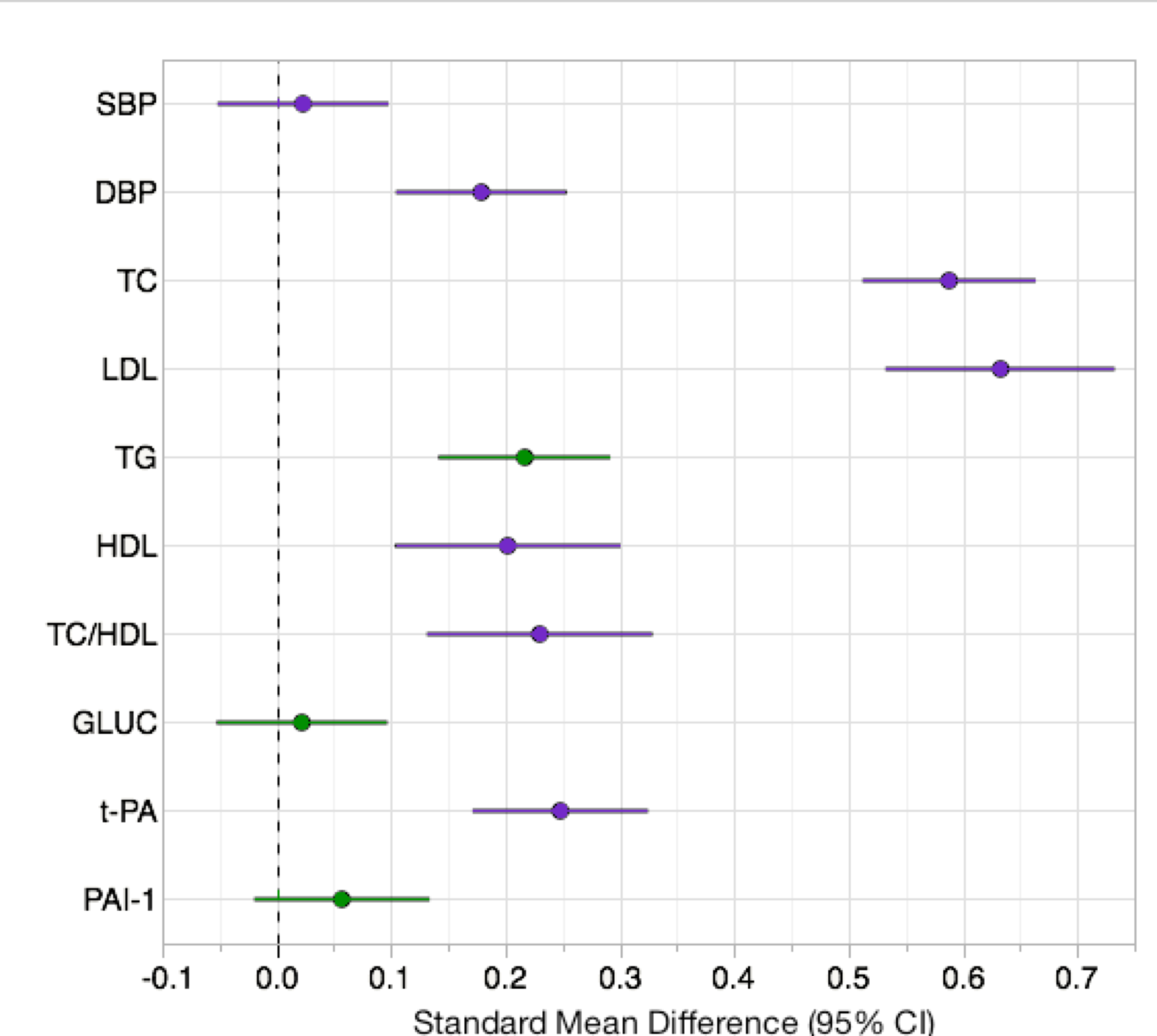
**

**Figure E.** **The BMI-adjusted effect of urban/rural environment on cardiovascular risk factors in Brong Ahafo, Ghana**. Absolute differences between urban and rural standardized means (with 95% confidence intervals) are depicted for each risk factor, with colors representing the group with the higher mean (purple: urban; green: rural). Data were adjusted for age, sex, and BMI.

**
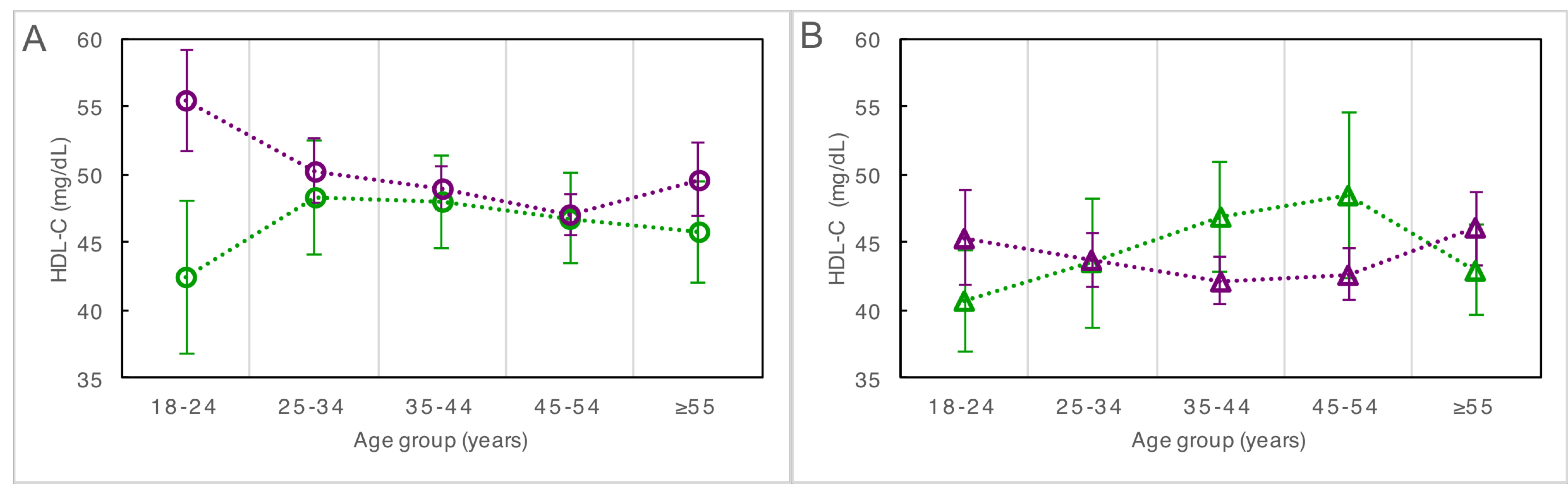
**

**Figure F.** **Mean high-density lipoprotein cholesterol by age group in urban and rural men and women in Brong Ahafo, Ghana**. (A) mean estimates by age group for urban females (purple circles) and rural females (green circles). (B) mean estimates by age group for urban males (purple triangles) and rural males (green triangles). Error bars denote 95% confidence intervals.

**
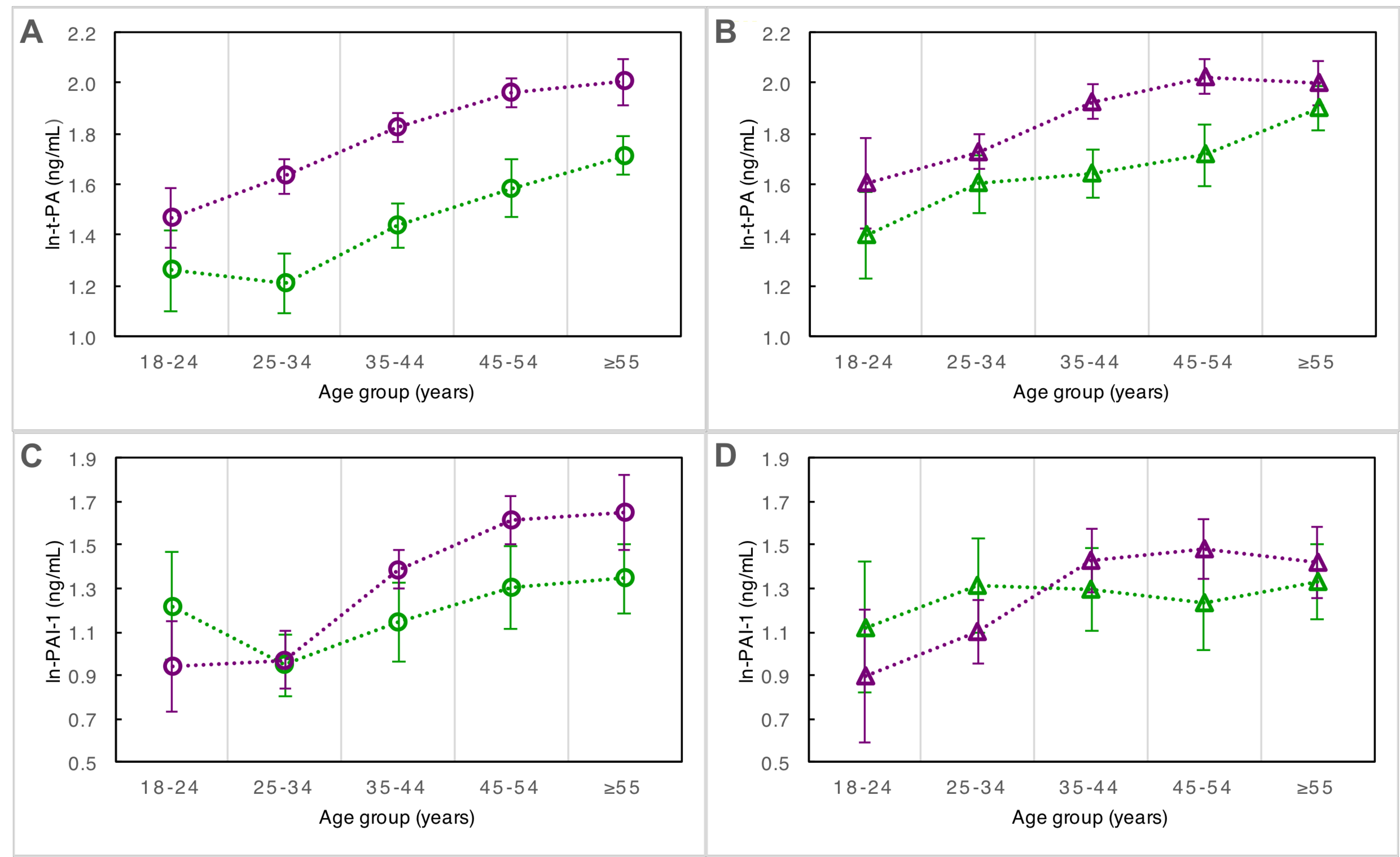
**

**Figure G.** **Mean t-PA and PAI-1 levels by age group in urban and rural men and women in Brong Ahafo, Ghana**. Left panels (A) and (C): urban females (purple circles) and rural females (green circles). Right panels (B) and (D): urban males (purple triangles) and rural males (green triangles). Error bars denote 95% confidence intervals.

**Table A.** Age-standardized prevalence rates and 95% confidence intervals of dichotomous risk factors in the Ghanaian cohort.

|  | **Females** | | | **Males** | | | **Urban** | **Rural** |
| --- | --- | --- | --- | --- | --- | --- | --- | --- |
|  | **Urban** | **Rural** | **p-value** | **Urban** | **Rural** | **p-value** | **p-value**  **by sex** | **p-value**  **by sex** |
| **N** | 1293 | 583 |  | 972 | 469 |  |  |  |
| **Hypertension** | 0.32 (0.30, 0.35) | 0.21  (0.18, 0.24) | <.001 | 0.34 (0.31, 0.37) | 0.20 (0.16, 0.24) | <.001 | 0.316 | 0.690 |
| **IFG (>100 mg/dL)** | 0.29  (0.27, 0.32) | 0.31  (0.28, 0.35) | 0.380 | 0.23 (0.20, 0.26) | 0.19 (0.16, 0.23) | 0.084 | 0.001 | <.001 |
| **IFG (>110 mg/dL)** | 0.12  (0.10, 0.14) | 0.10 (0.08, 0.13) | 0.207 | 0.10 (0.09, 0.12) | 0.05 (0.03, 0.07) | 0.001 | 0.133 | 0.003 |
| **Diabetes** | 0.07  (0.05, 0.08) | 0.03 (0.02, 0.05) | <.001 | 0.06 (0.04, 0.07) | 0.02 (0.01, 0.03) | <.001 | 0.342 | 0.307 |
| **Overweight** | 0.60  (0.58, 0.63) | 0.26 (0.22, 0.29) | <.001 | 0.35 (0.32, 0.38) | 0.11 (0.08, 0.14) | <.001 | <.001 | <.001 |
| **Obesity** | 0.26  (0.24, 0.28) | 0.05 (0.04, 0.07) | <.001 | 0.07 (0.05, 0.09) | 0.00 (0.00, 0.01) | <.001 | <.001 | <.001 |
| **Hypercholesterolemia** | 0.31  (0.28, 0.33) | 0.10 (0.08, 0.13) | <.001 | 0.22 (0.20, 0.25) | 0.07 (0.05, 0.09) | <.001 | <.001 | 0.086 |
| **High TG** | 0.21  (0.19, 0.24) | 0.26 (0.23, 0.30) | 0.017 | 0.28 (0.25, 0.31) | 0.27 (0.23, 0.31) | 0.691 | 0.001 | 0.715 |
| **Low HDL-C** | 0.26  (0.23, 0.29)^1^ | 0.38 (0.32, 0.43)^2^ | <.001 | 0.40 (0.36, 0.44)^3^ | 0.38 (0.32, 0.45)^4^ | 0.592 | <.001 | 0.946 |
| **High LDL-C** | 0.30  (0.27, 0.33)^1^ | 0.11 (0.08, 0.15)^2^ | <.001 | 0.22 (0.19, 0.25)^3^ | 0.05 (0.03, 0.08)^4^ | <.001 | <.001 | 0.014 |
| **Smoker** | 0.00  (0.00, 0.00) | 0.02 (0.01, 0.03) | <.001 | 0.03 (0.02, 0.04)) | 0.16 (0.13, 0.20) | <.001 | <.001 | <.001 |
| **Any schooling** | 0.88  (0.86, 0.90) | 0.44 (0.40, 0.48) | <.001 | 0.96 (0.95, 0.98) | 0.64 (0.60, 0.68) | <.001 | <.001 | <.001 |
| **Schooling >JSS** | 0.30 (0.27, 0.33) | 0.02 (0.01, 0.03) | <.001 | 0.48 (0.45, 0.52) | 0.05 (0.04, 0.08) | <.001 | <.001 | 0.007 |

^1^n=955, ^2^n=317, ^3^n=722, ^4^n=225

Hypertension: SBP ≥140 or DBP ≥90 or current use of medication; IFG: impaired fasting glucose; Diabetes: glucose ≥126 mg/dL or current use of medication; Overweight: BMI ≥25; Obese: BMI ≥30; Hypercholesterolemia: TC ≥200; High TG: TG ≥ 110 mg/dL; Low HDL-C: HDL ≤40 mg/dL; High LDL-C: LDL ≥130; Schooling >JSS: education beyond Junior Secondary School;

Prevalence age-standardized to WHO 2000-2025 standard population.
